# Association of HLA-DQA1*06:01 and DPB1*05:01:01G alleles with susceptibility to end-stage liver disease with HBV infection in liver transplant recipients from the Zhejiang Han population, China

**DOI:** 10.3389/fimmu.2025.1684437

**Published:** 2025-12-02

**Authors:** Genjie Lu, Yanmin He, Yangfang Lu, Wei Chen, Caide Lu, Faming Zhu

**Affiliations:** 1 Department of Blood Transfusion, Ningbo Medical Center Lihuili Hospital, The Affiliated Lihuili Hospital of Ningbo University, Ningbo, China; 2 HLA Typing Laboratory, Blood Center of Zhejiang Province, Hangzhou, China; 3 Department of Radiotherapy, Ningbo Medical Center Lihuili Hospital, The Affiliated Lihuili Hospital of Ningbo University, Ningbo, China; 4 Department of Hepatopancreatobiliary Surgery, Ningbo Medical Center Lihuili Hospital, The Affiliated Lihuili Hospital of Ningbo University, Ningbo, China

**Keywords:** end-stage liver disease, liver transplantation, human leukocyte antigen, next-generation sequencing, susceptibility

## Abstract

**Background:**

It has been reported that HLA was associated with susceptibility to various liver diseases. However, data on HLA in end-stage liver disease (ESLD) patients are limited. Therefore, this study aimed to explore the association of HLA with susceptibility to ESLD in patients undergoing liver transplantation (LT).

**Methods:**

108 ESLD patients who underwent LT from July 1, 2023, to December 31, 2024, were investigated. At the same time, 453 blood donors were randomly selected as healthy controls. Their specimens were genotyped for 11 HLA loci using next-generation sequencing (NGS).

**Results:**

There was no significant difference in gender and ABO blood group between the two groups (*P*>0.05) except that ESLD patients were older (*P* < 0.001). The allele frequencies (AFs) of HLA-B*18:02, -C*07:02, -DRB1*12:02, -DRB1*14:54, -DQA1*01:04, -DQA1*06:01, -DPA1*02:02, -DPB1*05:01:01G and -DPB1*135:01 in ESLD were significantly higher than those of the control group (*P* < 0.05), while the AFs of HLA-DRB1*15:01, -DQA1*01:01, -DQA1*01:02, -DQB1*06:02, -DPA1*01:03 and -DPB1*02:01:02G were significantly lower than that of the control group (*P* < 0.05). However, after correction by the step-down Bonferroni method, only HLA-DQA1*06:01 and HLA-DPB1*05:01:01G were considered statistically significant (*Pc* < 0.05). The association between these two HLA alleles and ESLD remained significant (*P* < 0.05) after adjustment for age and gender by binary logistic regression analysis. Subgroup analysis further revealed that this association was primarily linked to hepatitis B virus (HBV) infection. Compared with the control group, there were statistically significant differences in 3 haplotypes with frequencies >1.00% in ESLD (*P* < 0.05). However, none were statistically significant after correction (*Pc*>0.05).

**Conclusion:**

Our study suggested that HLA-DQA1*06:01 and HLA-DPB1*05:01:01G alleles may be associated with susceptibility to ESLD in liver transplant recipients, particularly in cases related to HBV infection.

## 1 Introduction

Liver disease causes two million deaths annually worldwide and accounts for 4% of all deaths (1). The concept of end-stage liver disease (ESLD) was first introduced in the 1980s, which refers to the end stage of chronic liver disease, with advanced liver injury, dysfunction, and decompensation, regardless of the etiology (2, 3). The main clinical feature of ESLD is the liver’s inability to meet the body’s physiological needs. The range of clinical disease forms of ESLD includes acute-on-chronic liver failure (ACLF), acute decompensated cirrhosis, chronic liver failure (CLF), and decompensated hepatocellular carcinoma (HCC) (4, 5). At present, liver transplantation (LT) is still the only way to cure ESLD (6). The model for end-stage liver disease (MELD) score is frequently used to assess the need for LT (7). Although LT is performed for cure ESLD in more than 100 countries worldwide, the majority of the patients with ESLD and life-threatening liver diseases are still unable to obtain a liver for transplantation (7, 8). Therefore, liver disease, especially ESLD, remains a considerable challenge.

The human leukocyte antigen (HLA) region is one of the most polymorphic genes in the human genome (9). HLA distribution differs significantly in various populations and ethnic groups (10, 11). With the rapid development of precise HLA genotyping technology, 42,193 HLA alleles have been identified in the latest IPD-IMGT/HLA database release 3.61version (2025–07) (12). HLA plays a critical role in human immunity. Class I molecules present endogenous peptides to CD8+ T cells, triggering cytotoxicity against infected cells. In contrast, class II molecules present exogenous antigens to activate T helper lymphocytes (13). In recent years, HLA has been reported to be associated with a variety of diseases (14–20), including viral hepatitis (14, 15), liver cancer (16), liver metabolic diseases (17, 18). However, the data for association of HLA with ESLD is still rare. Here, the association between HLA and ESLD in the Zhejiang Han population, China was studied.

## 2 Materials and methods

### 2.1 Study design and population

All patients with ESLD who underwent LT at Ningbo Medical Center Lihuili Hospital from July 1, 2023, to December 31, 2024 were enrolled. Inclusion criteria for the patients: (1)age ≥ 18 years; (2) diagnosis of ESLD requiring LT. In this study, the diagnosis of ESLD was primarily established by identifying at least one of the following conditions in the electronic medical records (EMR) admitting diagnoses: ACLF, decompensated cirrhosis, CLF, or decompensated HCC. These cases were subsequently confirmed as ESLD through a manual review conducted by our expert LT team. Exclusion criteria: (1) non-Zhejiang Han population; (2) a history of primary malignant tumors other than liver cancer; (3) autoimmune diseases other than autoimmune liver disease; (4) incomplete clinical data. A total of 108 patients with ESLD were finally enrolled in this study. At the same time, 453 healthy blood donors from the Zhejiang Han population in the same period were randomly selected as the control group. This study was approved by the ethics committee of Ningbo Medical Center Lihuili Hospital (Approval No: 2024 - 009). Written informed consent was obtained from all participants.

### 2.2 Data and specimen collection

Demographic and characteristics of ESLD patients and blood donors, including age, gender, ABO blood group were obtained through EMR or blood donation information systems, respectively. The remaining anticoagulant specimens for ABO blood group testing of ESLD patients and blood donors were collected respectively and stored below -20 °C until processing.

### 2.3 HLA genotyping

All specimens were genotyped for HLA-A, HLA-B, HLA-C, HLA-DRB1, HLA-DRB3, HLA-DRB4, HLA-DRB5, HLA-DQA1, HLA-DQB1, HLA-DPA1 and HLA-DPB1 using commerical AllType™NGS kit (One Lambda Inc, Canoga Park, CA, USA) and HLA genotype assignment was according to our previous reports (21–23).

### 2.4 Statistical analysis

Categorical variables were summarized as counts and percentages, with group comparisons performed using the chi-square test or Fisher’s exact test. Continuous variables exhibiting non-normal distributions were expressed as medians (interquartile ranges), and analyzed with the Mann-Whitney U test. The allele frequencies (AFs) and haplotype frequencies (HFs) of HLA loci, as well as Hardy–Weinberg equilibrium for each HLA locus, were determined by the Arlequin software 3.5.2.2 (24). Continuous variables such as age were dichotomized at the median. A binary logistic regression analysis model was used to predict ESLD, adjusted for specific HLA alleles, age, and gender. The *P* values of AFs and HFs were corrected by the step-down Bonferroni method (*Pc* values). A *P* value or *Pc* value less than 0.05 was considered statistically significant. Statistical calculation was performed by the SPSS statistical software, version 24.0 (IBM, Armonk, NY, USA).

## 3 Results

### 3.1 High hepatitis B virus infection rate was found in the ESLD patients

According to the EMR data, the ESLD patient group had a MELD score of 22.0 (19.0-24.0). Among them, 58 (53.70%) and 50 (46.30%) patients were graded as Child-Pugh B and C, respectively. Additionally, 77 cases (71.30%) were co-infected with the hepatitis B virus (HBV), which was also the most common underlying etiology of ESLD. There was no significant difference in gender and ABO blood group between the ESLD patient group and control group (*P*>0.05) except that ESLD patients were older (median age 52.5 years vs. 30.0 years) (*P* < 0.001). The demographic and ABO blood group distribution characteristics of the ESLD patients and blood donors were shown in **Table 1**.

**Table 1:** Basic characteristics of the ESLD patients and healthy controls in the Zhejiang Han population*.

Variables	ESLD (N = 108)	Control (N = 453)	*P* value
Median age (IQR), years	52.5 (47.0, 60.0)	30.0 (25.0, 38.0)	<0.001
Gender, n (%)			0.946
Female	28 (25.93)	116 (25.61)	
Male	80 (74.07)	337 (74.39)	
Blood group, n (%)			0.802
O	37 (34.26)	161 (35.54)	
Non O	71 (65.74)	292 (64.46)	
Underlying etiology, n (%)			
HBV	77 (71.30)	–	
HCV	2 (1.85)	–	
Alcoholic liver disease	8 (7.41)	–	
Autoimmune liver disease	14 (12.96)	–	
Inherited metabolic liver disease (Wilson disease)	1 (0.93)	–	
Nonalcoholic fatty liver disease	5 (4.63)	–	
Unknown	1 (0.93)	–	
MELD score	22.0 (19.0, 24.0)	–	
Child-Pugh classification, n (%)			
A	0 (0.00)	–	
B	58 (53.70)	–	
C	50 (46.30)	–	

*Data are shown as n (%) for categorical variables and median (interquartile range) for continuous variables.

### 3.2 AFs distribution of the 11 HLA loci in the ESLD patients and healthy controls

The allele distribution of the 11 HLA loci was fitted Hardy-Weinberg equilibrium (*P*>0.05) expect for HLA-DPA1 locus in the ESLD patients (**Supplementary Table S1**). However the *Pc* value was more than 0.05 for HLA-DPA1 locus. The frequencies of HLA alleles are summarized in **Supplementary Table S2**. The most frequent alleles of each locus in ESLD patients and healthy controls were HLA-A*11:01 (25.46% vs 22.74%), B*40:01(15.74% vs 12.36%), C*07:02(22.22% vs 15.56%), DRB1*12:02(15.74%) vs DRB1*09:01 (14.90%), DRB3*02:02(57.61% vs 56.73%), DRB4*01:03 (89.61% vs 91.82%), DRB5*01:01(80.00% vs 78.43%), DQA1*03:02 (16.20%) vs DQA1*01:02(17.66%), DQB1*03:01(26.39% vs 21.74%), DPA1*02:02 (57.87% vs 47.57%), DPB1*05:01:01G (49.07% vs 36.31%), respectively.

### 3.3 DQA1*06:01 and DPB1*05:01:01G may be associated with the susceptibility of ESLD

**Figure 1:**
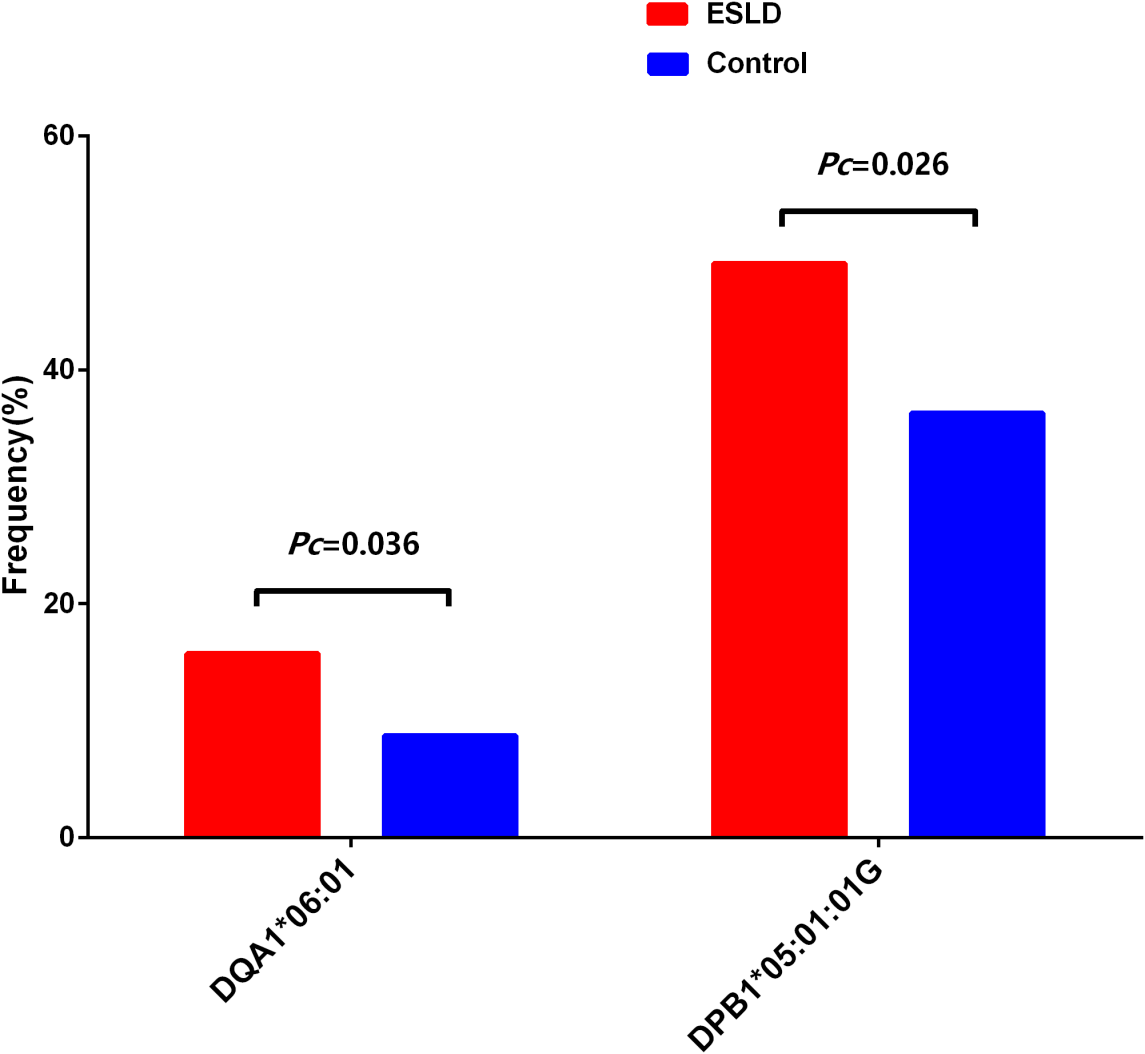
DQA1*06:01 and DPB1*05:01:01G may be associated with susceptibility to ESLD.

**Figure 2:**
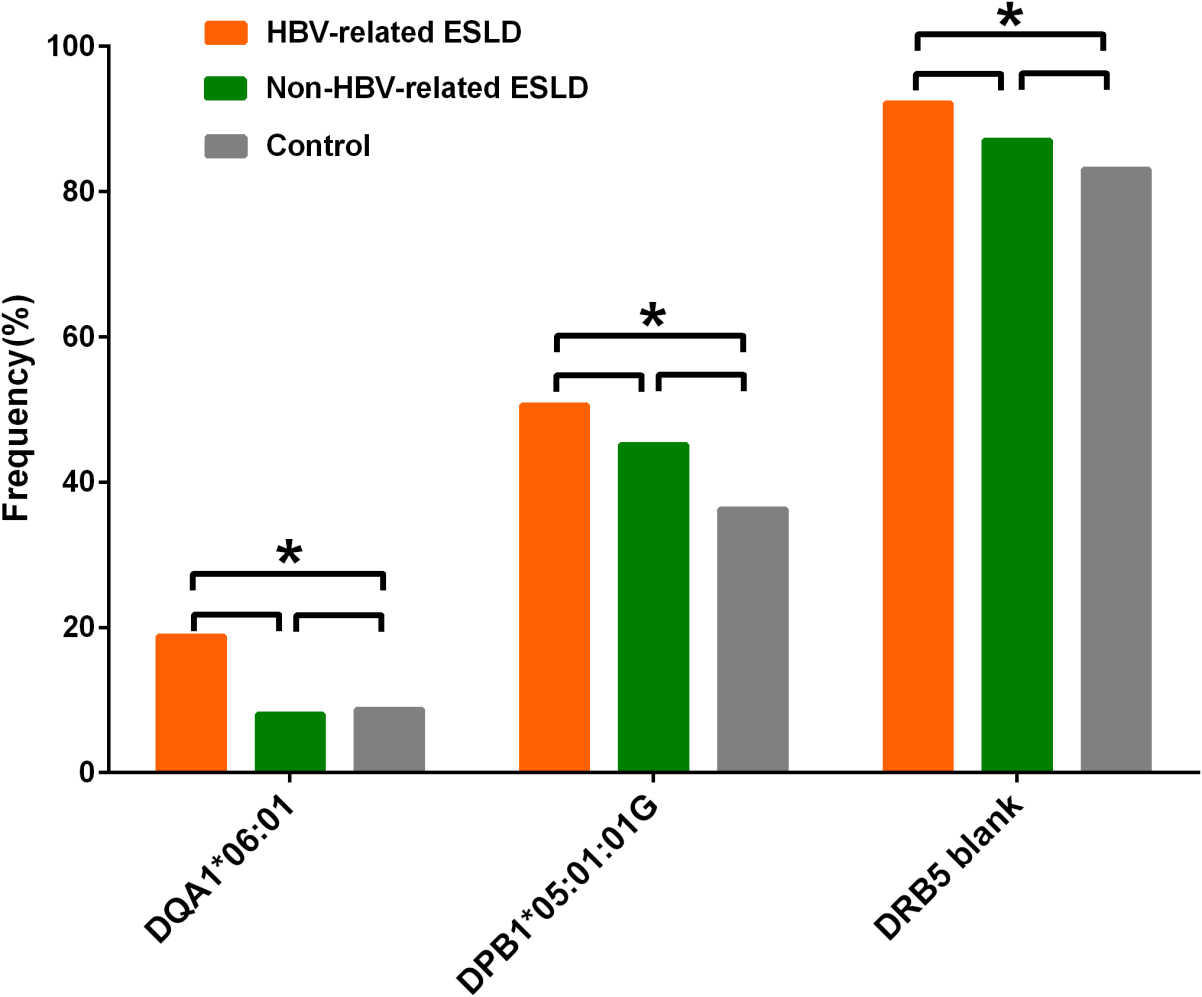
Pairwise comparisons of DQA1*06:01, DPB1*05:01:01G, and DRB5 blank were performed across the HBV-related ESLD group, the non-HBV-related ESLD group, and the control group. *Pc < 0.05 corrected by the step-down Bonferroni method.

15 HLA alleles of the HLA loci were showed uncorrected significant differences between the ESLD patient group and control group (**Table 2**). The AFs of HLA-B*18:02, -C*07:02, -DRB1*12:02, -DRB1*14:54, -DQA1*01:04, -DQA1*06:01, -DPA1*02:02, -DPB1*05:01:01G and -DPB1*135:01 in ESLD patient group were significantly higher than those of the control group (*P* < 0.05), while the AFs of HLA-DRB1*15:01, -DQA1*01:01, -DQA1*01:02, -DQB1*06:02, -DPA1*01:03 and -DPB1*02:01:02G were significantly lower than those of the control group (*P* < 0.05). However, only HLA-DQA1*06:01 and HLA-DPB1*05:01:01G were considered statistically significant (*Pc* < 0.05) after correction (**Figure 1**). Further it found that these associations were related to HBV infection in the subgroup analysis (**Figure 2** and **Supplementary Table S3**-**Supplementary Table S8**). However, in this subgroup of HBV-infected, no significant differences were observed in the frequencies of HLA-DQA1*06:01 and HLA-DPB1*05:01:01G between those with positive and negative in the pre-operative HBV DNA detection (*P*>0.05; **Supplementary Table S9**).

**Table 2:** HLA alleles with significant differences between ESLD patients and healthy controls in the Zhejiang Han population.

HLA allele	ESLD (N = 108)	Control (N = 453)	OR	95% CI for OR	*P* value	*Pc* value
	%	%				
B*18:02	0.93	0.00	–	–	0.037	>0.999
C*07:02	22.22	15.56	1.550	1.073-2.239	0.019	0.589
DRB1*12:02	15.74	8.61	1.983	1.285-3.059	0.002	0.084
DRB1*14:54	5.09	1.99	2.647	1.231-5.690	0.010	0.400
DRB1*15:01	6.02	12.25	0.459	0.253-0.831	0.009	0.369
DQA1*01:01	0.93	3.53	0.255	0.061-1.073	0.045	0.675
DQA1*01:02	11.11	17.66	0.583	0.369-0.921	0.019	0.304
DQA1*01:04	8.33	4.19	2.077	1.161-3.715	0.012	0.204
DQA1*06:01	15.74	8.72	1.956	1.269-3.015	0.002	0.036
DQB1*06:02	3.70	9.27	0.376	0.179-0.790	0.007	0.119
DPA1*01:03	25.93	34.55	0.663	0.475-0.926	0.015	0.105
DPA1*02:02	57.87	47.57	1.514	1.122-2.043	0.007	0.056
DPB1*02:01:02G	12.04	18.32	0.610	0.392-0.950	0.028	0.672
DPB1*05:01:01G	49.07	36.31	1.690	1.253-2.279	0.001	0.026
DPB1*135:01	1.39	0.00	–	–	0.007	0.175

OR , odds ratio; CI , confidence interval.

### 3.4 HLA-DRB5 blank may be associated with the susceptibility of ESLD

HLA-DRB5 blank frequency was higher in the ESLD patients than that in the control group (90.74% vs. 83.11%, *P* = 0.005, *Pc* = 0.035) (**Supplementary Table S10**). Similarly, The proportion of two copy of HLA-DRB5 absence in the ESLD patients was higher than that in the control group (82.4% vs. 68.9%, *P* = 0.005). However, this phenomenon was not found in HLA-DRB3 or HLA-DRB4 loci. Further subgroup analysis found that the association was related to HBV infection (**Figure 2** and **Supplementary Table S11**-**Supplementary Table S13**). However, no significant differences of HLA-DRB5 blank frequencies were observed between those with positive and negative in the pre-operative HBV DNA detection (*P*>0.05; **Supplementary Table S9**).

### 3.5 Associations persisted after adjustment for age and gender

**Figure 3:**
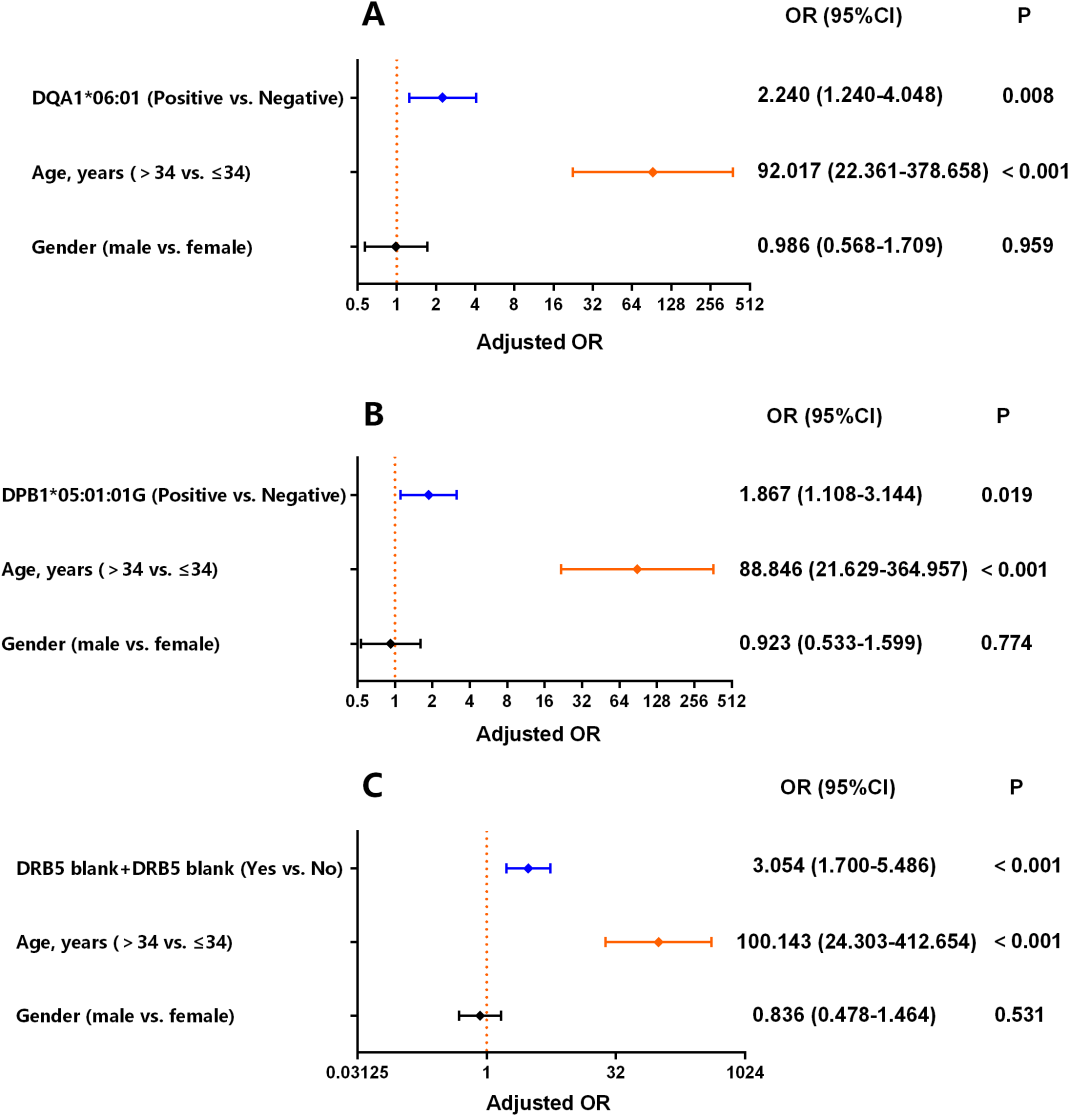
Binary logistic regression for predicting ESLD, adjusted for specific HLA alleles, age, and gender. **(A)** adjusted for HLA-DQA1*06:01; **(B)** adjusted for HLA-DPB1*05:01:01G; **(C)** adjusted for the absence of HLA-DRB5.

In the binary logistic regression analysis adjusted for age and gender, HLA-DQA1*06:01, -DPB1*05:01:01G, and the two copy of HLA-DRB5 absence remained independent risk factors for ESLD (*P* < 0.05; **Figure 3**). Additionally, among the healthy controls, no significant difference was observed in the frequencies of HLA-DQA1*06:01, -DPB1*05:01:01G, and -DRB5 blank between the age >30 and age ≤30 subgroups (*Pc*>0.05; **Supplementary Table S14**).

### 3.6 DQA1*06:01, DPB1*05:01:01G, and DRB5 blank may not be associated with ESLD severity

In patients with ESLD, the frequencies of HLA-DQA1*06:01, -DPB1*05:01:01G, and -DRB5 blank did not differ significantly between groups with MELD scores >22 and ≤22 (*P*>0.05; **Table 3**). Similarly, no significant differences in their frequencies were observed between patients with Child-Pugh grade B and grade C (*P*>0.05; **Table 4**).

**Table 3:** Comparison of HLA AFs between MELD score groups (>22 vs ≤22) in ESLD patients from the Zhejiang Han population.

HLA allele	MELD score > 22 (N = 38)	MELD score ≤ 22 (N = 70)	OR	95% CI for OR	*P*-value	*Pc*-value
	%	%				
DQA1*06:01	15.79	15.71	1.006	0.467-2.164	0.988	>0.999
DPB1*05:01:01G	46.05	50.71	0.830	0.475-1.452	0.513	>0.999
DRB5 blank	93.42	89.29	1.704	0.594-4.885	0.317	>0.999

**Table 4:** Comparison of HLA AFs across Child-Pugh grades B and C in ESLD patients from the Zhejiang Han population.

HLA allele	Child-Pugh grade C (N = 50)	Child-Pugh grade B (N = 58)	OR	95% CI for OR	*P*-value	*Pc*-value
	%	%				
DQA1*06:01	12.00	18.97	0.583	0.272-1.247	0.161	>0.999
DPB1*05:01:01G	44.00	53.45	0.684	0.400-1.171	0.166	>0.999
DRB5 blank	93.00	88.79	1.677	0.642-4.382	0.288	>0.999

### 3.7 No HLA haplotypes association with ESLD patients

A total of 182 and 651 different HLA haplotypes were estimated in the ESLD patient group and control group. The highest frequencies in the ESLD patient group were A*11:01-C*07:02-B*40:01-DRB1*12:02-DQA1*06:01-DQB1*03:01-DPA1*02:02-DPB1*05:01:01G (2.31%) and A*30:01-C*06:02-B*13:02-DRB1*07:01-DQA1*02:01-DQB1*02:02-DPA1*02:01-DPB1*17:01:01G (2.31%), while the latter also had the highest frequency in the control group (2.76%). HLA haplotypes with frequencies >1% were listed in the **Table 5**. A total of 3 haplotypes were found to have uncorrected significant differences (*P* < 0.05), but none had substantial differences after correction (*Pc*>0.05).

**Table 5:** Comparison of the 8 HLA loci haplotypes with frequency > 1% between ESLD and healthy controls in the Zhejiang Han population.

HLA haplotype	ESLD (%)	Control (%)	*P* value	*Pc* value
A*11:01-C*07:02-B*40:01-DRB1*12:02-DQA1*06:01-DQB1*03:01-DPA1*02:02-DPB1*05:01:01G	2.31	0.22	0.002	>0.999
A*30:01-C*06:02-B*13:02-DRB1*07:01-DQA1*02:01-DQB1*02:02-DPA1*02:01-DPB1*17:01:01G	2.31	2.76	0.873	>0.999
A*02:07-C*01:02-B*46:01-DRB1*12:02-DQA1*06:01-DQB1*03:01-DPA1*02:02-DPB1*05:01:01G	1.85	0.11	0.035	>0.999
A*33:03-C*03:02-B*58:01-DRB1*03:01-DQA1*05:01-DQB1*02:01-DPA1*01:03-DPB1*04:01:01G	1.85	2.06	>0.999	>0.999
A*02:01-C*03:04-B*13:01-DRB1*09:01:02G-DQA1*03:02-DQB1*03:03-DPA1*02:02-DPB1*05:01:01G	1.39	0.33	0.073	>0.999
A*11:01-C*07:02-B*40:01-DRB1*09:01:02G-DQA1*03:02-DQB1*03:03-DPA1*02:02-DPB1*05:01:01G	1.39	0.11	0.035	>0.999
A*02:07-C*01:02-B*46:01-DRB1*09:01:02G-DQA1*03:02-DQB1*03:03-DPA1*02:02-DPB1*05:01:01G	0.00	2.20	0.095	>0.999
A*30:01-C*06:02-B*13:02-DRB1*07:01-DQA1*02:01-DQB1*02:02-DPA1*02:02-DPB1*05:01:01G	0.93	1.32	>0.999	>0.999
A*11:01-C*07:02-B*40:01-DRB1*08:03-DQA1*01:03-DQB1*06:01-DPA1*02:02-DPB1*05:01:01G	0.00	1.31	0.234	>0.999

## 4 Discussion

In the healthy controls of this study, the frequencies of HLA-DQA1*06:01 and -DPB1*05:01:01G were observed to be 8.72% and 36.31%, respectively, which are consistent with the previously reported frequencies of 7.87% and 39.79% in the local population (22). Meanwhile, among the HLA-DRB5 alleles, DRB5*01:01 showed the highest frequency at 78.43%, also aligning with the previously reported 77.26% in the local population (22). However, the study did not specify the frequency of HLA-DRB5 blank (22). In our study, the frequency of HLA-DRB5 blank was 83.11%, which is close to the 87.31% reported in the neighboring country of Korea (11). Furthermore, to minimize potential population stratification bias, the Hardy–Weinberg equilibrium test was performed.

There are many studies on the association between HLA and susceptibility to liver diseases, especially viral hepatitis (25–27). However, there are limited data on the association between HLA and susceptibility to ESLD. Tillmann et al. (28) found that HLA-DRB1*11 and HLA-DQB1*03 were significantly lower in hepatitis C virus (HCV) induced ESLD patients. However, it was not found in our study. This difference may be due to the different etiologies of ESLD in the two studies, which were mainly induced by HCV and HBV, respectively, and the difference in HLA distribution in the various populations.

The main etiologies of ESLD in north America are alcohol-related liver disease, non-alcoholic steatohepatitis (NASH), and chronic HCV (29). However, it is mainly associated with chronic HBV infection in Asian populations (30). Most of liver transplant recipients had chronic HBV infection in this study. It was found that HLA-DQA1*06:01 and HLA-DPB1*05:01:01G may be associated with susceptibility in ESLD in this study. However, we did not find any association between these HLA alleles and the severity of ESLD. Studies (27, 31) have reported that HLA-DQA1*06:01 may be a predisposing factor for HBV infection and progression to cirrhosis. Additional studies (25, 32) have reported HLA-DPB1*05:01 as a risk factor for HBV infection. Since about 70% of ESLD in our study were also chronically infected with HBV, therefore HLA-DQA1*06:01 and HLA-DPB1*05:01:01G as risk factors for ESLD may be original for HBV infection, which was validated in our subgroup analysis. In the HBV-infected subgroup, we further assessed the impact of the patients’ infection status by comparing those with detectable and undetectable pre-operative HBV DNA levels. However, no significant differences in HLA alleles were observed between these two groups. Furthermore, given that the vast majority of HBV-infected ESLD patients had received antiviral therapy prior to surgery (70/77) and had comorbid cirrhosis (74/77), these factors were not considered as potential sources of bias in the comparative analysis.

However, the mechanisms underlying these associations are not yet fully understood. HLA class II molecules play a critical role in shaping CD4+ T lymphocyte responses via antigen presentation. Notably, the HLA-DQ molecule is distinct from other class II antigens because most of its variable amino acid residues reside within the α-helix region of the antigen-binding groove (33). Thus, we hypothesize that the association of HLA-DQB1*06:01 with chronic HBV infection could stem from its reduced capacity to efficiently bind and present HBV antigens. Nevertheless, this hypothesis requires validation through further investigation. In addition, a proposed mechanism for the association of HLA-DPB1*05:01:01G with HBV infection involves its reduced mRNA expression, which could impair antigen presentation (25). This impairment may subsequently diminish the immune response against HBV, thereby increasing susceptibility to persistent infection. An additional theory suggests that HLA-DPB1*05:01 exhibits suboptimal binding affinity for specific peptide epitopes derived from HBV nucleocapsid proteins, leading to a reduced likelihood of viral clearance (34).

HLA-DQA1*01:02 has been suggested to be a protective factor for HBV infection (31, 35), but it was not found in this study. Previous studies (25, 26, 32) have found that HLA-DPB1*02:01 is a protective factor for HBV infection, and it was also observed in our study of the ESLD patients, but it was not statistically significant after adjustment. HLA-DPB1*09:01 has been reported (25, 26) as a risk factor for HBV infection but was not found in ESLD in this study. The reason need to further explore and one of them may be due to about 30% of ESLD patients with not HBV-infected in this study.

Interestingly, HLA-DRB5 blank was found as risk factors for ESLD patients. It has been reported that a lack of HLA-DRB5 may be associated with various diseases, including rheumatoid arthritis patients with negative antibodies against citrullinated peptides (36) and patients with multiple sclerosis (37). Therefore, the absence of HLA-DRB5 may require more attention and further research in the future.

HLA AFs may differ across age groups (38). In our study, blood donors were used as controls. While this selection may result in a “healthier” control group, the significant age difference between the younger blood donors and the older ESLD patients raised the concern that the observed differences in the frequencies of HLA-DQA1*06:01, -DPB1*05:01:01G, and -DRB5 blank might be attributable to age variation rather than disease association. To address this, we stratified the control group by median age into younger and older subgroups. However, no significant differences in the frequencies of these key HLA alleles were observed between the two subgroups. Most importantly, we performed logistic regression adjusted for age and gender as confounding factors. The results demonstrated that the associations between these HLA alleles and ESLD remained significant, thereby strengthening the reliability of our findings.

We observed significantly elevated frequencies of HLA-DQA1*06:01, -DPB1*05:01:01G, and -DRB5 blank in patients with ESLD. This finding not only carries population genetic relevance but also implies potential clinical importance for immune risk management after LT. Firstly, HLA-DQ mismatch is a well-established risk factor for *de novo* donor-specific antibody (DSA) development, and anti-DQ antibodies are strongly associated with antibody-mediated rejection and graft loss (39). The high frequency of HLA-DQA1*06:01 in our cohort suggests that these recipients may harbor a genetic background predisposing them to heightened immune responses against mismatched donor HLA-DQ antigens. Secondly, in hematopoietic stem cell transplantation, the immunologic impact of HLA-DPB1 is clearly defined: non-permissive mismatches potently enhance alloreactive T-cell activation, increasing the incidence of acute graft-versus-host disease and mortality (40). Although evidence in LT remains limited, the underlying principles of alloimmunity are likely shared. Thus, HLA-DPB1*05:01:01G mismatch may elevate the risk of T-cell-mediated rejection and adversely affect long-term graft survival via cytotoxic T-lymphocyte pathways. Finally, the absence of DRB5 in a recipient implies that donor DRB5 will be recognized as a foreign antigen, substantially increasing the risk of developing anti-DRB5 DSAs. In solid organ transplantation, antibodies against HLA class II antigens are powerful predictors of chronic rejection (41). Therefore, implementing high-resolution matching at these loci before transplantation and evaluating the permissibility of mismatches could improve donor-recipient selection and potentially enhance post-transplant outcomes.

There are several limitations in this study. Firstly, the collection of indicators in ESLD and healthy controls, such as body mass index (BMI), was incomplete. Secondly, the sample number was relatively small, especially for ESLD. Therefore, a multicenter study with a larger sample size is warranted to validate our findings further. Thirdly, the control group was significantly younger than the study group. These differences may lead to misinterpretation of the results because HLA alleles may differ between young and old populations. Thus, it needs to be studied further in the future.

## 5 Conclusion

In this study, the association between AFs and HFs of 11 HLA loci at high resolution level and ESLD was firstly analyzed in the Zhejiang Han population, China. It was found that HLA-DQA1*06:01, HLA-DPB1*05:01:01G and HLA-DRB5 absence may be associated with susceptibility to ESLD in liver transplant recipients, particularly in cases related to HBV infection. These results will help further understand the role of HLA loci in the pathogenesis of ESLD requiring LT in China.

## Data Availability

The original contributions presented in the study are included in the article/**Supplementary Material**, further inquiries can be directed to the corresponding authors.
