# Supplementary material for: Association of HLA-DQA1*06:01 and DPB1*05:01:01G alleles with susceptibility to end-stage liver disease with HBV infection in liver transplant recipients from the Zhejiang Han population, China
Source: Front Immunol. 2025 Dec 2;16:1684437. doi: 10.3389/fimmu.2025.1684437 (PMC12705408; doi:10.3389/fimmu.2025.1684437)
Supplement: Supplementary file 1 [file DataSheet1.docx]

Table S1. Hardy-Weinberg equilibrium of the 11 HLA loci between the ESLD and healthy controls.

| Locus | Heterozygosity in the ESLD group (N=108) | | | Heterozygosity in the healthy control group (N=453) | | |
| --- | --- | --- | --- | --- | --- | --- |
|  | Observed | Expected | *P* value | Observed | Expected | *P* value |
| HLA-A | 0.84259 | 0.87196 | 0.277 | 0.90287 | 0.88492 | 0.641 |
| HLA-B | 0.90741 | 0.94143 | 0.122 | 0.93819 | 0.95120 | 0.107 |
| HLA-C | 0.82407 | 0.88889 | 0.046 | 0.90728 | 0.91223 | 0.084 |
| HLA-DRB1 | 0.90741 | 0.92166 | 0.052 | 0.91832 | 0.92465 | 0.759 |
| HLA-DRB3 | 0.61111 | 0.59100 | 0.269 | 0.54305 | 0.51957 | 0.477 |
| HLA-DRB4 | 0.52778 | 0.48557 | 0.505 | 0.49448 | 0.48415 | 0.246 |
| HLA-DRB5 | 0.17593 | 0.17179 | 0.361 | 0.29139 | 0.29154 | 0.802 |
| HLA-DQA1 | 0.89815 | 0.90164 | 0.111 | 0.88079 | 0.89680 | 0.524 |
| HLA-DQB1 | 0.88889 | 0.86985 | 0.096 | 0.88079 | 0.88551 | 0.086 |
| HLA-DPA1 | 0.53704 | 0.58880 | 0.007 | 0.63135 | 0.63637 | 0.369 |
| HLA-DPB1 | 0.70370 | 0.73131 | 0.411 | 0.79470 | 0.81120 | 0.442 |

Table S2 Comparison of all alleles of 11 HLA loci between ESLD (n=108) and healthy controls (n=453)

| Alleles | Frequency of ESLD (%) | Frequency of healthy controls (%) | *P*-value | OR | 95% CI for OR | *Pc*-value |
| --- | --- | --- | --- | --- | --- | --- |
| A*01:01 | 1.85 | 1.55 | 0.983 | 1.202 | 0.392-3.689 | >0.999 |
| A*02:01 | 13.89 | 13.69 | 0.938 | 1.017 | 0.662-1.563 | >0.999 |
| A*02:03 | 4.63 | 3.31 | 0.348 | 1.417 | 0.682-2.946 | >0.999 |
| A*02:05 | 0.93 | 0.22 | 0.169 | 4.224 | 0.592-30.159 | >0.999 |
| A*02:06 | 3.24 | 5.19 | 0.230 | 0.612 | 0.273-1.374 | >0.999 |
| A*02:07 | 7.87 | 8.61 | 0.726 | 0.907 | 0.525-1.567 | >0.999 |
| A*02:10 | 0.00 | 0.44 | >0.999 | - | - | >0.999 |
| A*03:01 | 1.39 | 2.43 | 0.501 | 0.566 | 0.168-1.908 | >0.999 |
| A*03:02 | 0.00 | 0.11 | >0.999 | - | - | >0.999 |
| A*11:01 | 25.46 | 22.74 | 0.394 | 1.161 | 0.824-1.636 | >0.999 |
| A*11:02 | 2.78 | 1.99 | 0.645 | 1.410 | 0.553-3.594 | >0.999 |
| A*11:03 | 0.00 | 0.11 | >0.999 | - | - | >0.999 |
| A*24:02 | 14.81 | 14.24 | 0.828 | 1.048 | 0.689-1.593 | >0.999 |
| A*24:03 | 0.00 | 0.33 | >0.999 | - | - | >0.999 |
| A*24:07 | 0.00 | 0.11 | >0.999 | - | - | >0.999 |
| A*24:10 | 0.93 | 0.11 | 0.097 | 8.458 | 0.763-93.708 | >0.999 |
| A*24:20 | 0.00 | 0.22 | >0.999 | - | - | >0.999 |
| A*26:01 | 2.78 | 2.10 | 0.724 | 1.334 | 0.526-3.381 | >0.999 |
| A*29:01 | 0.46 | 0.77 | 0.971 | 0.597 | 0.073-4.881 | >0.999 |
| A*29:02 | 0.00 | 0.22 | >0.999 | - | - | >0.999 |
| A*30:01 | 4.63 | 6.73 | 0.254 | 0.672 | 0.339-1.335 | >0.999 |
| A*30:02 | 0.00 | 0.22 | >0.999 | - | - | >0.999 |
| A*30:18 | 0.00 | 0.11 | >0.999 | - | - | >0.999 |
| A*31:01 | 2.78 | 2.98 | 0.874 | 0.930 | 0.379-2.282 | >0.999 |
| A*31:17 | 0.00 | 0.11 | >0.999 | - | - | >0.999 |
| A*32:01 | 0.93 | 1.88 | 0.497 | 0.489 | 0.112-2.131 | >0.999 |
| A*33:01 | 0.00 | 0.22 | >0.999 | - | - | >0.999 |
| A*33:03 | 10.65 | 8.28 | 0.268 | 1.320 | 0.807-2.161 | >0.999 |
| A*68:01 | 0.00 | 0.55 | 0.590 | - | - | >0.999 |
| A*69:01 | 0.00 | 0.44 | >0.999 | - | - | >0.999 |
| B*07:02 | 1.85 | 1.55 | 0.983 | 1.202 | 0.392-3.689 | >0.999 |
| B*07:05 | 0.46 | 0.77 | 0.971 | 0.597 | 0.073-4.881 | >0.999 |
| B*08:01 | 0.46 | 0.33 | 0.575 | 1.400 | 0.145-13.525 | >0.999 |
| B*13:01 | 6.48 | 4.64 | 0.263 | 1.426 | 0.764-2.661 | >0.999 |
| B*13:02 | 4.63 | 7.40 | 0.149 | 0.608 | 0.307-1.202 | >0.999 |
| B*14:02 | 0.00 | 0.22 | >0.999 | - | - | >0.999 |
| B*15:01 | 4.63 | 3.86 | 0.606 | 1.208 | 0.589-2.479 | >0.999 |
| B*15:02 | 3.24 | 3.64 | 0.775 | 0.886 | 0.387-2.031 | >0.999 |
| B*15:07 | 0.46 | 0.44 | >0.999 | 1.049 | 0.117-9.431 | >0.999 |
| B*15:11 | 0.46 | 1.99 | 0.205 | 0.229 | 0.030-1.728 | >0.999 |
| B*15:12 | 0.46 | 0.22 | 0.474 | 2.102 | 0.190-23.292 | >0.999 |
| B*15:13 | 0.46 | 0.00 | 0.193 | - | - | >0.999 |
| B*15:17 | 0.00 | 0.22 | >0.999 | - | - | >0.999 |
| B*15:18 | 0.00 | 1.88 | 0.086 | - | - | >0.999 |
| B*15:19 | 0.00 | 0.11 | >0.999 | - | - | >0.999 |
| B*15:25 | 0.46 | 0.55 | >0.999 | 0.838 | 0.097-7.211 | >0.999 |
| B*15:27 | 0.46 | 1.32 | 0.478 | 0.347 | 0.045-2.679 | >0.999 |
| B*15:58 | 0.00 | 0.11 | >0.999 | - | - | >0.999 |
| B*15:68 | 0.00 | 0.11 | >0.999 | - | - | >0.999 |
| B*18:01 | 0.00 | 0.66 | 0.603 | - | - | >0.999 |
| B*18:02 | 0.93 | 0.00 | 0.037 | - | - | >0.999 |
| B*27:04 | 0.93 | 1.43 | 0.798 | 0.642 | 0.144-2.866 | >0.999 |
| B*27:05 | 0.93 | 0.66 | >0.999 | 1.402 | 0.281-6.994 | >0.999 |
| B*27:07 | 0.00 | 0.11 | >0.999 | - | - | >0.999 |
| B*27:24 | 0.00 | 0.11 | >0.999 | - | - | >0.999 |
| B*35:01 | 3.24 | 2.54 | 0.565 | 1.286 | 0.544-3.037 | >0.999 |
| B*35:02 | 0.00 | 0.11 | >0.999 | - | - | >0.999 |
| B*35:03 | 0.46 | 0.99 | 0.732 | 0.464 | 0.058-3.679 | >0.999 |
| B*35:05 | 0.00 | 0.22 | >0.999 | - | - | >0.999 |
| B*37:01 | 1.39 | 0.77 | 0.643 | 1.809 | 0.464-7.053 | >0.999 |
| B*38:01 | 0.46 | 0.44 | >0.999 | 1.049 | 0.117-9.431 | >0.999 |
| B*38:02 | 4.17 | 1.99 | 0.060 | 2.145 | 0.950-4.843 | >0.999 |
| B*39:01 | 2.78 | 2.21 | 0.617 | 1.266 | 0.502-3.191 | >0.999 |
| B*39:05 | 0.00 | 0.33 | >0.999 | - | - | >0.999 |
| B*39:15 | 0.00 | 0.11 | >0.999 | - | - | >0.999 |
| B*39:24 | 0.00 | 0.11 | >0.999 | - | - | >0.999 |
| B*40:01 | 15.74 | 12.36 | 0.185 | 1.324 | 0.873-2.008 | >0.999 |
| B*40:02 | 2.31 | 2.54 | 0.850 | 0.910 | 0.342-2.421 | >0.999 |
| B*40:03 | 0.93 | 0.22 | 0.169 | 4.224 | 0.592-30.159 | >0.999 |
| B*40:06 | 2.31 | 2.65 | 0.781 | 0.871 | 0.328-2.309 | >0.999 |
| B*41:01 | 0.00 | 0.33 | >0.999 | - | - | >0.999 |
| B*44:02 | 0.00 | 0.33 | >0.999 | - | - | >0.999 |
| B*44:03 | 2.78 | 3.09 | 0.810 | 0.896 | 0.366-2.191 | >0.999 |
| B*45:01 | 0.00 | 0.33 | >0.999 | - | - | >0.999 |
| B*46:01 | 11.11 | 9.49 | 0.472 | 1.192 | 0.738-1.924 | >0.999 |
| B*47:01 | 0.00 | 0.11 | >0.999 | - | - | >0.999 |
| B*48:01 | 1.85 | 1.77 | >0.999 | 1.050 | 0.347-3.171 | >0.999 |
| B*48:03 | 0.46 | 0.22 | 0.474 | 2.102 | 0.190-23.292 | >0.999 |
| B*50:01 | 0.46 | 0.44 | >0.999 | 1.049 | 0.117-9.431 | >0.999 |
| B*51:01 | 3.70 | 4.75 | 0.509 | 0.772 | 0.358-1.667 | >0.999 |
| B*51:02 | 1.39 | 1.21 | >0.999 | 1.146 | 0.317-4.144 | >0.999 |
| B*51:07 | 0.00 | 0.11 | >0.999 | - | - | >0.999 |
| B*51:08 | 0.00 | 0.11 | >0.999 | - | - | >0.999 |
| B*52:01 | 0.93 | 3.20 | 0.067 | 0.283 | 0.067-1.194 | >0.999 |
| B*53:01 | 0.00 | 0.22 | >0.999 | - | - | >0.999 |
| B*54:01 | 1.85 | 3.42 | 0.233 | 0.533 | 0.186-1.525 | >0.999 |
| B*55:01 | 0.46 | 0.00 | 0.193 | - | - | >0.999 |
| B*55:02 | 2.78 | 2.43 | 0.767 | 1.148 | 0.460-2.867 | >0.999 |
| B*55:04 | 0.00 | 0.11 | >0.999 | - | - | >0.999 |
| B*55:12 | 0.46 | 0.00 | 0.193 | - | - | >0.999 |
| B*55:91 | 0.00 | 0.11 | >0.999 | - | - | >0.999 |
| B*56:01 | 0.46 | 0.33 | 0.575 | 1.400 | 0.145-13.525 | >0.999 |
| B*56:04 | 0.00 | 0.11 | >0.999 | - | - | >0.999 |
| B*57:01 | 0.46 | 0.55 | >0.999 | 0.838 | 0.097-7.211 | >0.999 |
| B*58:01 | 7.87 | 6.18 | 0.366 | 1.297 | 0.738-2.280 | >0.999 |
| B*67:01 | 1.85 | 0.66 | 0.205 | 2.830 | 0.792-10.118 | >0.999 |
| B*81:02 | 0.00 | 0.44 | >0.999 | - | - | >0.999 |
| C*01:02 | 16.67 | 15.23 | 0.601 | 1.113 | 0.745-1.662 | >0.999 |
| C*01:03 | 0.00 | 0.77 | 0.415 | - | - | >0.999 |
| C*02:02 | 0.93 | 0.77 | >0.999 | 1.200 | 0.248-5.819 | >0.999 |
| C*03:02 | 8.33 | 6.29 | 0.280 | 1.354 | 0.780-2.352 | >0.999 |
| C*03:03 | 5.09 | 6.84 | 0.349 | 0.730 | 0.378-1.412 | >0.999 |
| C*03:04 | 11.57 | 9.16 | 0.280 | 1.298 | 0.808-2.085 | >0.999 |
| C*04:01 | 4.63 | 4.86 | 0.889 | 0.951 | 0.471-1.921 | >0.999 |
| C*04:03 | 0.93 | 0.88 | >0.999 | 1.049 | 0.221-4.976 | >0.999 |
| C*04:06 | 0.00 | 0.11 | >0.999 | - | - | >0.999 |
| C*04:82 | 0.00 | 0.55 | 0.590 | - | - | >0.999 |
| C*05:01 | 0.00 | 0.22 | >0.999 | - | - | >0.999 |
| C*06:02 | 6.94 | 9.60 | 0.222 | 0.703 | 0.398-1.241 | >0.999 |
| C*07:01 | 0.00 | 0.77 | 0.415 | - | - | >0.999 |
| C*07:02 | 22.22 | 15.56 | 0.019 | 1.550 | 1.073-2.239 | 0.589 |
| C*07:04 | 0.93 | 1.32 | 0.894 | 0.696 | 0.155-3.134 | >0.999 |
| C*07:06 | 0.46 | 0.99 | 0.732 | 0.464 | 0.058-3.679 | >0.999 |
| C*07:63 | 0.00 | 0.11 | >0.999 | - | - | >0.999 |
| C*07:66 | 0.46 | 0.11 | 0.348 | 4.209 | 0.262-67.565 | >0.999 |
| C*08:01 | 6.48 | 8.50 | 0.329 | 0.746 | 0.414-1.346 | >0.999 |
| C*08:02 | 0.00 | 0.22 | >0.999 | - | - | >0.999 |
| C*08:03 | 0.00 | 0.44 | >0.999 | - | - | >0.999 |
| C*08:22 | 0.93 | 1.10 | >0.999 | 0.837 | 0.182-3.850 | >0.999 |
| C*12:02 | 1.85 | 3.75 | 0.165 | 0.484 | 0.170-1.378 | >0.999 |
| C*12:03 | 1.85 | 1.43 | 0.888 | 1.296 | 0.418-4.015 | >0.999 |
| C*14:02 | 2.78 | 3.97 | 0.405 | 0.690 | 0.287-1.660 | >0.999 |
| C*14:03 | 1.85 | 1.43 | 0.888 | 1.296 | 0.418-4.015 | >0.999 |
| C*15:02 | 4.17 | 3.64 | 0.715 | 1.150 | 0.542-2.441 | >0.999 |
| C*15:04 | 0.00 | 0.11 | >0.999 | - | - | >0.999 |
| C*15:05 | 0.46 | 0.77 | 0.971 | 0.597 | 0.073-4.881 | >0.999 |
| C*16:02 | 0.46 | 0.11 | 0.348 | 4.209 | 0.262-67.565 | >0.999 |
| C*17:01 | 0.00 | 0.33 | >0.999 | - | - | >0.999 |
| DRB1*01:01 | 0.46 | 2.43 | 0.118 | - | - | >0.999 |
| DRB1*01:02 | 0.00 | 0.22 | >0.999 | - | - | >0.999 |
| DRB1*03:01 | 4.63 | 5.63 | 0.560 | 0.814 | 0.406-1.630 | >0.999 |
| DRB1*04:01 | 1.39 | 1.10 | >0.999 | 1.262 | 0.344-4.625 | >0.999 |
| DRB1*04:02 | 0.00 | 0.22 | >0.999 | - | - | >0.999 |
| DRB1*04:03 | 2.31 | 1.55 | 0.621 | 1.510 | 0.538-4.238 | >0.999 |
| DRB1*04:04 | 0.00 | 1.10 | 0.251 | - | - | >0.999 |
| DRB1*04:05 | 4.63 | 4.53 | 0.947 | 1.024 | 0.505-2.078 | >0.999 |
| DRB1*04:06 | 2.31 | 2.43 | 0.922 | 0.952 | 0.356-2.544 | >0.999 |
| DRB1*04:07 | 0.00 | 0.22 | >0.999 | - | - | >0.999 |
| DRB1*04:08 | 0.00 | 0.33 | >0.999 | - | - | >0.999 |
| DRB1*04:10 | 0.46 | 0.00 | 0.193 | - | - | >0.999 |
| DRB1*07:01 | 8.80 | 9.93 | 0.612 | 0.874 | 0.521-1.469 | >0.999 |
| DRB1*07:13 | 0.00 | 0.11 | >0.999 | - | - | >0.999 |
| DRB1*08:02 | 0.93 | 0.55 | 0.883 | 1.684 | 0.325-8.739 | >0.999 |
| DRB1*08:03 | 8.33 | 7.40 | 0.640 | 1.138 | 0.661-1.959 | >0.999 |
| DRB1*08:09 | 0.46 | 0.22 | 0.474 | 2.102 | 0.190-23.292 | >0.999 |
| DRB1*09:01 | 15.28 | 14.90 | 0.889 | 1.030 | 0.681-1.557 | >0.999 |
| DRB1*09:14 | 0.46 | 0.00 | 0.193 | - | - | >0.999 |
| DRB1*10:01 | 2.31 | 1.32 | 0.447 | 1.765 | 0.615-5.065 | >0.999 |
| DRB1*11:01 | 5.09 | 7.51 | 0.213 | 0.661 | 0.343-1.273 | >0.999 |
| DRB1*11:04 | 0.00 | 0.11 | >0.999 | - | - | >0.999 |
| DRB1*11:06 | 0.00 | 0.11 | >0.999 | - | - | >0.999 |
| DRB1*11:245 | 0.00 | 0.11 | >0.999 | - | - | >0.999 |
| DRB1*12:01:01G | 4.17 | 3.42 | 0.596 | 1.227 | 0.575-2.617 | >0.999 |
| DRB1*12:02 | 15.74 | 8.61 | 0.002 | 1.983 | 1.285-3.059 | 0.080 |
| DRB1*12:08 | 0.00 | 0.11 | >0.999 | - | - | >0.999 |
| DRB1*13:01 | 0.00 | 0.77 | 0.415 | - | - | >0.999 |
| DRB1*13:02 | 2.78 | 3.09 | 0.810 | 0.896 | 0.366-2.191 | >0.999 |
| DRB1*13:07 | 0.00 | 0.11 | >0.999 | - | - | >0.999 |
| DRB1*13:12 | 0.00 | 0.33 | >0.999 | - | - | >0.999 |
| DRB1*14:03 | 0.46 | 0.22 | 0.474 | 2.102 | 0.190-23.292 | >0.999 |
| DRB1*14:04 | 0.00 | 0.66 | 0.603 | - | - | >0.999 |
| DRB1*14:05 | 3.70 | 1.43 | 0.053 | 2.642 | 1.081-6.457 | >0.999 |
| DRB1*14:18 | 0.46 | 0.22 | 0.474 | 2.102 | 0.190-23.292 | >0.999 |
| DRB1*14:25 | 0.46 | 0.00 | 0.193 | - | - | >0.999 |
| DRB1*14:54 | 5.09 | 1.99 | 0.010 | 2.647 | 1.231-5.690 | 0.380 |
| DRB1*15:01 | 6.02 | 12.25 | 0.009 | 0.459 | 0.253-0.831 | 0.351 |
| DRB1*15:02:01G | 0.46 | 2.54 | 0.102 | 0.179 | 0.024-1.330 | >0.999 |
| DRB1*16:02 | 2.78 | 2.21 | 0.617 | 1.266 | 0.502-3.191 | >0.999 |
| DRB3*01:01 | 8.70 | 12.18 | 0.355 | 0.687 | 0.308-1.529 | >0.999 |
| DRB3*02:02 | 57.61 | 56.73 | 0.881 | 1.037 | 0.648-1.659 | >0.999 |
| DRB3*03:01 | 33.70 | 30.77 | 0.595 | 1.143 | 0.697-1.875 | >0.999 |
| DRB3*03:65 | 0.00 | 0.32 | >0.999 | - | - | >0.999 |
| DRB4*01:01 | 2.60 | 1.21 | 0.702 | 2.173 | 0.391-12.087 | >0.999 |
| DRB4*01:02 | 2.60 | 3.03 | >0.999 | 0.853 | 0.183-3.976 | >0.999 |
| DRB4*01:03 | 89.61 | 91.82 | 0.534 | 0.769 | 0.335-1.765 | >0.999 |
| DRB4*01:03N | 5.19 | 3.94 | 0.858 | 1.336 | 0.423-4.216 | >0.999 |
| DRB5*01:01 | 80.00 | 78.43 | >0.999 | 1.1 | 0.344-3.514 | >0.999 |
| DRB5*01:02 | 0.00 | 11.11 | 0.242 | - | - | >0.999 |
| DRB5*01:03 | 5.00 | 1.31 | 0.31 | 3.974 | 0.344-45.929 | >0.999 |
| DRB5*01:08N | 0.00 | 1.31 | >0.999 | - | - | >0.999 |
| DRB5*02:02 | 5.00 | 5.88 | >0.999 | 0.842 | 0.101-7.020 | >0.999 |
| DRB5*02:03 | 10.00 | 1.96 | 0.103 | 5.556 | 0.869-35.502 | 0.412 |
| DQA1*01:01 | 0.93 | 3.53 | 0.045 | 0.255 | 0.061-1.073 | 0.675 |
| DQA1*01:02 | 11.11 | 17.66 | 0.019 | 0.583 | 0.369-0.921 | 0.304 |
| DQA1*01:03 | 8.80 | 9.60 | 0.716 | 0.908 | 0.540-1.527 | >0.999 |
| DQA1*01:04 | 8.33 | 4.19 | 0.012 | 2.077 | 1.161-3.715 | 0.204 |
| DQA1*01:05 | 2.31 | 1.32 | 0.447 | 1.765 | 0.615-5.065 | >0.999 |
| DQA1*01:09 | 0.46 | 0.00 | 0.193 | - | - | >0.999 |
| DQA1*02:01 | 8.80 | 10.04 | 0.579 | 0.864 | 0.514-1.450 | >0.999 |
| DQA1*03:01 | 5.09 | 5.52 | 0.804 | 0.919 | 0.470-1.796 | >0.999 |
| DQA1*03:02 | 16.20 | 15.12 | 0.692 | 1.085 | 0.724-1.627 | >0.999 |
| DQA1*03:03 | 6.48 | 5.96 | 0.773 | 1.094 | 0.596-2.008 | >0.999 |
| DQA1*04:01 | 0.93 | 0.88 | >0.999 | 1.049 | 0.221-4.976 | >0.999 |
| DQA1*05:01 | 4.63 | 5.63 | 0.560 | 0.814 | 0.406-1.630 | >0.999 |
| DQA1*05:03 | 0.46 | 0.66 | >0.999 | 0.698 | 0.084-5.825 | >0.999 |
| DQA1*05:05 | 8.33 | 10.04 | 0.446 | 0.814 | 0.480-1.382 | >0.999 |
| DQA1*05:06 | 0.46 | 0.11 | 0.348 | 4.209 | 0.262-67.565 | >0.999 |
| DQA1*05:08 | 0.93 | 0.77 | >0.999 | 1.200 | 0.248-5.819 | >0.999 |
| DQA1*05:09 | 0.00 | 0.22 | >0.999 | - | - | >0.999 |
| DQA1*06:01 | 15.74 | 8.72 | 0.002 | 1.956 | 1.269-3.015 | 0.036 |
| DQB1*02:01 | 4.63 | 5.63 | 0.560 | 0.814 | 0.406-1.630 | >0.999 |
| DQB1*02:02 | 6.94 | 8.61 | 0.425 | 0.792 | 0.446-1.406 | >0.999 |
| DQB1*03:01 | 26.39 | 21.74 | 0.143 | 1.290 | 0.917-1.815 | >0.999 |
| DQB1*03:02 | 5.56 | 5.41 | 0.932 | 1.029 | 0.537-1.970 | >0.999 |
| DQB1*03:03 | 17.59 | 16.56 | 0.714 | 1.076 | 0.727-1.592 | >0.999 |
| DQB1*03:05 | 0.00 | 0.11 | >0.999 | - | - | >0.999 |
| DQB1*03:13 | 0.46 | 0.11 | 0.348 | 4.209 | 0.262-67.565 | >0.999 |
| DQB1*04:01 | 5.09 | 4.53 | 0.722 | 1.132 | 0.572-2.241 | >0.999 |
| DQB1*04:02 | 1.39 | 0.99 | 0.889 | 1.404 | 0.377-5.229 | >0.999 |
| DQB1*05:01 | 2.78 | 4.64 | 0.225 | 0.588 | 0.247-1.401 | >0.999 |
| DQB1*05:02 | 6.48 | 3.97 | 0.108 | 1.675 | 0.887-3.164 | >0.999 |
| DQB1*05:03 | 6.02 | 4.42 | 0.318 | 1.386 | 0.728-2.640 | >0.999 |
| DQB1*06:01 | 10.19 | 10.15 | 0.989 | 1.003 | 0.614-1.639 | >0.999 |
| DQB1*06:02 | 3.70 | 9.27 | 0.007 | 0.376 | 0.179-0.790 | 0.119 |
| DQB1*06:03 | 0.00 | 0.77 | 0.415 | - | - | >0.999 |
| DQB1*06:04 | 1.39 | 1.43 | >0.999 | 0.967 | 0.273-3.425 | >0.999 |
| DQB1*06:09 | 1.39 | 1.66 | >0.999 | 0.837 | 0.240-2.916 | >0.999 |
| DPA1*01:03 | 25.93 | 34.55 | 0.015 | 0.663 | 0.475-0.926 | 0.105 |
| DPA1*01:04 | 0.00 | 0.11 | >0.999 | - | - | >0.999 |
| DPA1*02:01 | 10.19 | 13.25 | 0.224 | 0.743 | 0.459-1.202 | >0.999 |
| DPA1*02:02 | 57.87 | 47.57 | 0.007 | 1.514 | 1.122-2.043 | 0.056 |
| DPA1*02:06 | 0.46 | 0.00 | 0.193 | - | - | >0.999 |
| DPA1*02:07 | 1.85 | 1.55 | 0.983 | 1.202 | 0.392-3.689 | >0.999 |
| DPA1*02:10 | 0.46 | 0.00 | 0.193 | - | - | >0.999 |
| DPA1*04:01 | 3.24 | 2.98 | 0.841 | 1.090 | 0.468-2.538 | >0.999 |
| DPB1*01:01:01G | 0.00 | 0.11 | >0.999 | - | - | >0.999 |
| DPB1*02:01:02G | 12.04 | 18.32 | 0.028 | 0.610 | 0.392-0.950 | 0.672 |
| DPB1*02:02:01G | 6.48 | 6.95 | 0.805 | 0.927 | 0.509-1.688 | >0.999 |
| DPB1*03:01 | 0.46 | 0.00 | 0.193 | - | - | >0.999 |
| DPB1*03:01:01G | 3.70 | 4.08 | 0.798 | 0.903 | 0.414-1.969 | >0.999 |
| DPB1*04:01:01G | 7.41 | 9.49 | 0.338 | 0.763 | 0.438-1.329 | >0.999 |
| DPB1*04:02:01G | 3.70 | 4.42 | 0.642 | 0.833 | 0.384-1.806 | >0.999 |
| DPB1*05:01:01G | 49.07 | 36.31 | 0.001 | 1.690 | 1.253-2.279 | 0.026 |
| DPB1*09:01:01G | 1.39 | 1.55 | >0.999 | 0.897 | 0.256-3.151 | >0.999 |
| DPB1*10:01:01G | 0.46 | 0.44 | >0.999 | 1.049 | 0.117-9.431 | >0.999 |
| DPB1*104:01 | 0.00 | 0.11 | >0.999 | - | - | >0.999 |
| DPB1*13:01:01G | 4.17 | 5.30 | 0.496 | 0.777 | 0.375-1.609 | >0.999 |
| DPB1*135:01 | 1.39 | 0.00 | 0.007 | - | - | 0.175 |
| DPB1*14:01:01G | 3.24 | 3.64 | 0.775 | 0.886 | 0.387-2.031 | >0.999 |
| DPB1*15:01:01G | 0.00 | 0.11 | >0.999 | - | - | >0.999 |
| DPB1*17:01:01G | 2.78 | 4.30 | 0.304 | 0.635 | 0.265-1.520 | >0.999 |
| DPB1*19:01:01G | 1.39 | 1.21 | >0.999 | 1.146 | 0.317-4.144 | >0.999 |
| DPB1*21:01:01G | 0.93 | 1.88 | 0.497 | 0.489 | 0.112-2.131 | >0.999 |
| DPB1*26:01:02G | 0.46 | 0.33 | 0.575 | 1.400 | 0.145-13.525 | >0.999 |
| DPB1*28:01:01G | 0.46 | 0.22 | 0.474 | 2.102 | 0.190-23.292 | >0.999 |
| DPB1*31:01:01G | 0.00 | 0.22 | >0.999 | - | - | >0.999 |
| DPB1*34:01:01G | 0.00 | 0.11 | >0.999 | - | - | >0.999 |
| DPB1*38:01:01G | 0.00 | 0.33 | >0.999 | - | - | >0.999 |
| DPB1*41:01:01G | 0.00 | 0.33 | >0.999 | - | - | >0.999 |
| DPB1*47:01:01G | 0.46 | 0.11 | 0.348 | 4.209 | 0.262-67.565 | >0.999 |
| DPB1*93:01 | 0.00 | 0.11 | >0.999 | - | - | >0.999 |

Table S3 Comparison of all HLA-DQA1 alleles between HBV-related ESLD (n=77) and healthy controls (n=453) in the Zhejiang Han population.

| Alleles | Frequency of HBV-related ESLD (%) | Frequency of healthy controls (%) | *P*-value | OR | 95% CI for OR | *Pc*-value |
| --- | --- | --- | --- | --- | --- | --- |
| DQA1*01:01 | 1.30 | 3.53 | 0.228 | 0.359 | 0.085-1.515 | >0.999 |
| DQA1*01:02 | 9.09 | 17.66 | 0.008 | 0.466 | 0.262-0.829 | 0.136 |
| DQA1*01:03 | 8.44 | 9.60 | 0.649 | 0.868 | 0.472-1.597 | >0.999 |
| DQA1*01:04 | 7.79 | 4.19 | 0.052 | 1.93 | 0.985-3.783 | 0.832 |
| DQA1*01:05 | 3.25 | 1.32 | 0.159 | 2.5 | 0.868-7.199 | >0.999 |
| DQA1*01:09 | 0.65 | 0.00 | 0.145 | - | - | >0.999 |
| DQA1*02:01 | 9.09 | 10.04 | 0.714 | 0.896 | 0.496-1.617 | >0.999 |
| DQA1*03:01 | 3.90 | 5.52 | 0.405 | 0.694 | 0.292-1.648 | >0.999 |
| DQA1*03:02 | 15.58 | 15.12 | 0.882 | 1.036 | 0.646-1.661 | >0.999 |
| DQA1*03:03 | 5.19 | 5.96 | 0.708 | 0.865 | 0.403-1.854 | >0.999 |
| DQA1*04:01 | 1.30 | 0.88 | 0.966 | 1.477 | 0.311-7.022 | >0.999 |
| DQA1*05:01 | 4.55 | 5.63 | 0.585 | 0.798 | 0.355-1.793 | >0.999 |
| DQA1*05:03 | 0.00 | 0.66 | 0.601 | - | - | >0.999 |
| DQA1*05:05 | 9.74 | 10.04 | 0.907 | 0.966 | 0.544-1.718 | >0.999 |
| DQA1*05:06 | 0.65 | 0.11 | 0.270 | 5.915 | 0.368-95.068 | >0.999 |
| DQA1*05:08 | 0.65 | 0.77 | >0.999 | 0.839 | 0.103-6.870 | >0.999 |
| DQA1*05:09 | 0.00 | 0.22 | >0.999 | - | - | >0.999 |
| DQA1*06:01 | 18.83 | 8.72 | ＜0.001 | 2.429 | 1.525-3.867 | 0.002 |

Table S4 Comparison of all HLA-DQA1 alleles between non-HBV-related ESLD (n=31) and healthy controls (n=453) in the Zhejiang Han population.

| Alleles | Frequency of non-HBV-related ESLD (%) | Frequency of healthy controls (%) | *P*-value | OR | 95% CI for OR | *Pc*-value |
| --- | --- | --- | --- | --- | --- | --- |
| DQA1*01:01 | 0.00 | 3.53 | 0.255 | - | - | >0.999 |
| DQA1*01:02 | 16.13 | 17.66 | 0.759 | 0.897 | 0.446-1.802 | >0.999 |
| DQA1*01:03 | 9.68 | 9.60 | 0.985 | 1.009 | 0.422-2.409 | >0.999 |
| DQA1*01:04 | 9.68 | 4.19 | 0.091 | 2.477 | 0.993-6.034 | >0.999 |
| DQA1*01:05 | 0.00 | 1.32 | >0.999 | - | - | >0.999 |
| DQA1*02:01 | 8.06 | 10.04 | 0.614 | 0.786 | 0.307-2.010 | >0.999 |
| DQA1*03:01 | 8.06 | 5.52 | 0.579 | 1.502 | 0.576-3.913 | >0.999 |
| DQA1*03:02 | 17.74 | 15.12 | 0.579 | 1.211 | 0.616-2.381 | >0.999 |
| DQA1*03:03 | 9.68 | 5.96 | 0.367 | 1.690 | 0.697-4.099 | >0.999 |
| DQA1*04:01 | 0.00 | 0.88 | >0.999 | - | - | >0.999 |
| DQA1*05:01 | 4.84 | 5.63 | >0.999 | 0.852 | 0.258-2.813 | >0.999 |
| DQA1*05:03 | 1.61 | 0.66 | 0.372 | 2.459 | 0.291-20.751 | >0.999 |
| DQA1*05:05 | 4.84 | 10.04 | 0.181 | 0.455 | 0.140-1.482 | >0.999 |
| DQA1*05:06 | 0.00 | 0.11 | >0.999 | - | - | >0.999 |
| DQA1*05:08 | 1.61 | 0.77 | 0.412 | 2.105 | 0.255-17.388 | >0.999 |
| DQA1*05:09 | 0.00 | 0.22 | >0.999 | - | - | >0.999 |
| DQA1*06:01 | 8.06 | 8.72 | 0.859 | 0.918 | 0.358-2.357 | >0.999 |

Table S5 Comparison of all HLA-DQA1 alleles between HBV-related ESLD (n=77) and non-HBV-related ESLD (n=31) in the Zhejiang Han population.

| Alleles | Frequency of HBV-related ESLD (%) | Frequency of non-HBV-related ESLD (%) | *P*-value | OR | 95% CI for OR | *Pc*-value |
| --- | --- | --- | --- | --- | --- | --- |
| DQA1*01:01 | 1.30 | 0.00 | >0.999 | - | - | >0.999 |
| DQA1*01:02 | 9.09 | 16.13 | 0.136 | 0.520 | 0.217-1.243 | >0.999 |
| DQA1*01:03 | 8.44 | 9.68 | 0.772 | 0.861 | 0.312-2.376 | >0.999 |
| DQA1*01:04 | 7.79 | 9.68 | 0.650 | 0.789 | 0.282-2.204 | >0.999 |
| DQA1*01:05 | 3.25 | 0.00 | 0.325 | - | - | >0.999 |
| DQA1*01:09 | 0.65 | 0.00 | >0.999 | - | - | >0.999 |
| DQA1*02:01 | 9.09 | 8.06 | 0.810 | 1.140 | 0.392-3.312 | >0.999 |
| DQA1*03:01 | 3.90 | 8.06 | 0.358 | 0.462 | 0.136-1.574 | >0.999 |
| DQA1*03:02 | 15.58 | 17.74 | 0.697 | 0.856 | 0.391-1.874 | >0.999 |
| DQA1*03:03 | 5.19 | 9.68 | 0.365 | 0.511 | 0.170-1.540 | >0.999 |
| DQA1*04:01 | 1.30 | 0.00 | >0.999 | - | - | >0.999 |
| DQA1*05:01 | 4.55 | 4.84 | >0.999 | 0.937 | 0.234-3.744 | >0.999 |
| DQA1*05:03 | 0.00 | 1.61 | 0.287 | - | - | >0.999 |
| DQA1*05:05 | 9.74 | 4.84 | 0.238 | 2.122 | 0.592-7.606 | >0.999 |
| DQA1*05:06 | 0.65 | 0.00 | >0.999 | - | - | >0.999 |
| DQA1*05:08 | 0.65 | 1.61 | 0.493 | 0.399 | 0.025-6.476 | >0.999 |
| DQA1*06:01 | 18.83 | 8.06 | 0.049 | 2.645 | 0.974-7.185 | 0.833 |

Table S6 Comparison of all HLA-DPB1 alleles between HBV-related ESLD (n=77) and healthy controls (n=453) in the Zhejiang Han population.

| Alleles | Frequency of HBV-related ESLD (%) | Frequency of healthy controls (%) | *P*-value | OR | 95% CI for OR | *Pc*-value |
| --- | --- | --- | --- | --- | --- | --- |
| DPB1*01:01:01G | 0.00 | 0.11 | >0.999 | - | - | >0.999 |
| DPB1*02:01:02G | 7.79 | 18.32 | 0.001 | 0.377 | 0.204-0.695 | 0.030 |
| DPB1*02:02:01G | 5.19 | 6.95 | 0.420 | 0.733 | 0.344-1.562 | >0.999 |
| DPB1*03:01:01G | 3.90 | 4.08 | 0.913 | 0.952 | 0.395-2.296 | >0.999 |
| DPB1*04:01:01G | 9.74 | 9.49 | 0.923 | 1.029 | 0.578-1.832 | >0.999 |
| DPB1*04:02:01G | 4.55 | 4.42 | 0.942 | 1.031 | 0.453-2.345 | >0.999 |
| DPB1*05:01:01G | 50.65 | 36.31 | 0.001 | 1.800 | 1.276-2.538 | 0.018 |
| DPB1*09:01:01G | 1.30 | 1.55 | >0.999 | 0.838 | 0.189-3.726 | >0.999 |
| DPB1*10:01:01G | 0.00 | 0.44 | >0.999 | - | - | >0.999 |
| DPB1*104:01 | 0.00 | 0.11 | >0.999 | - | - | >0.999 |
| DPB1*13:01:01G | 5.84 | 5.30 | 0.781 | 1.109 | 0.533-2.310 | >0.999 |
| DPB1*135:01 | 1.30 | 0.00 | 0.021 | - | - | 0.483 |
| DPB1*14:01:01G | 2.60 | 3.64 | 0.514 | 0.705 | 0.246-2.020 | >0.999 |
| DPB1*15:01:01G | 0.00 | 0.11 | >0.999 | - | - | >0.999 |
| DPB1*17:01:01G | 2.60 | 4.30 | 0.321 | 0.593 | 0.209-1.683 | >0.999 |
| DPB1*19:01:01G | 1.30 | 1.21 | >0.999 | 1.071 | 0.235-4.877 | >0.999 |
| DPB1*21:01:01G | 1.30 | 1.88 | 0.864 | 0.688 | 0.257-3.008 | >0.999 |
| DPB1*26:01:02G | 0.65 | 0.33 | 0.467 | 1.967 | 0.203-19.036 | >0.999 |
| DPB1*28:01:01G | 0.65 | 0.22 | 0.376 | 2.954 | 0.266-32.780 | >0.999 |
| DPB1*31:01:01G | 0.00 | 0.22 | >0.999 | - | - | >0.999 |
| DPB1*34:01:01G | 0.00 | 0.11 | >0.999 | - | - | >0.999 |
| DPB1*38:01:01G | 0.00 | 0.33 | >0.999 | - | - | >0.999 |
| DPB1*41:01:01G | 0.00 | 0.33 | >0.999 | - | - | >0.999 |
| DPB1*47:01:01G | 0.65 | 0.11 | 0.270 | 5.915 | 0.368-95.068 | >0.999 |
| DPB1*93:01 | 0.00 | 0.11 | >0.999 | - | - | >0.999 |

Table S7 Comparison of all HLA-DPB1 alleles between non-HBV-related ESLD (n=31) and healthy controls (n=453) in the Zhejiang Han population.

| Alleles | Frequency of non-HBV-related ESLD (%) | Frequency of healthy controls (%) | *P*-value | OR | 95% CI for OR | *Pc*-value |
| --- | --- | --- | --- | --- | --- | --- |
| DPB1*01:01:01G | 0.00 | 0.11 | >0.999 | - | - | >0.999 |
| DPB1*02:01:02G | 22.58 | 18.32 | 0.404 | 1.300 | 0.700-2.414 | >0.999 |
| DPB1*02:02:01G | 9.68 | 6.95 | 0.581 | 1.434 | 0.595-3.456 | >0.999 |
| DPB1*03:01 | 1.61 | 0.00 | 0.064 | - | - | >0.999 |
| DPB1*03:01:01G | 3.23 | 4.08 | >0.999 | 0.783 | 0.184-3.327 | >0.999 |
| DPB1*04:01:01G | 1.61 | 9.49 | 0.036 | 0.156 | 0.021-1.142 | 0.936 |
| DPB1*04:02:01G | 1.61 | 4.42 | 0.463 | 0.355 | 0.048-2.626 | >0.999 |
| DPB1*05:01:01G | 45.16 | 36.31 | 0.162 | 1.444 | 0.860-2.425 | >0.999 |
| DPB1*09:01:01G | 1.61 | 1.55 | >0.999 | 1.044 | 0.135-8.075 | >0.999 |
| DPB1*10:01:01G | 1.61 | 0.44 | 0.282 | 3.697 | 0.407-33.584 | >0.999 |
| DPB1*104:01 | 0.00 | 0.11 | >0.999 | - | - | >0.999 |
| DPB1*13:01:01G | 0.00 | 5.30 | 0.120 | - | - | >0.999 |
| DPB1*135:01 | 1.61 | 0.00 | 0.064 | - | - | >0.999 |
| DPB1*14:01:01G | 4.84 | 3.64 | 0.893 | 1.345 | 0.401-4.515 | >0.999 |
| DPB1*15:01:01G | 0.00 | 0.11 | >0.999 | - | - | >0.999 |
| DPB1*17:01:01G | 3.23 | 4.30 | 0.935 | 0.741 | 0.175-3.143 | >0.999 |
| DPB1*19:01:01G | 1.61 | 1.21 | >0.999 | 1.334 | 0.169-10.502 | >0.999 |
| DPB1*21:01:01G | 0.00 | 1.88 | 0.556 | - | - | >0.999 |
| DPB1*26:01:02G | 0.00 | 0.33 | >0.999 | - | - | >0.999 |
| DPB1*28:01:01G | 0.00 | 0.22 | >0.999 | - | - | >0.999 |
| DPB1*31:01:01G | 0.00 | 0.22 | >0.999 | - | - | >0.999 |
| DPB1*34:01:01G | 0.00 | 0.11 | >0.999 | - | - | >0.999 |
| DPB1*38:01:01G | 0.00 | 0.33 | >0.999 | - | - | >0.999 |
| DPB1*41:01:01G | 0.00 | 0.33 | >0.999 | - | - | >0.999 |
| DPB1*47:01:01G | 0.00 | 0.11 | >0.999 | - | - | >0.999 |
| DPB1*93:01 | 0.00 | 0.11 | >0.999 | - | - | >0.999 |

Table S8 Comparison of all HLA-DPB1 alleles between HBV-related ESLD (n=77) and non-HBV-related ESLD (n=31) in the Zhejiang Han population.

| Alleles | Frequency of HBV-related ESLD (%) | Frequency of non-HBV-related ESLD (%) | *P*-value | OR | 95% CI for OR | *Pc*-value |
| --- | --- | --- | --- | --- | --- | --- |
| DPB1*02:01:02G | 7.79 | 22.58 | 0.003 | 0.290 | 0.125-0.670 | 0.045 |
| DPB1*02:02:01G | 5.19 | 9.68 | 0.365 | 0.511 | 0.170-1.540 | >0.999 |
| DPB1*03:01 | 0.00 | 1.61 | 0.287 | - | - | >0.999 |
| DPB1*03:01:01G | 3.90 | 3.23 | >0.999 | 1.216 | 0.239-6.196 | >0.999 |
| DPB1*04:01:01G | 9.74 | 1.61 | 0.076 | 6.583 | 0.850-50.955 | >0.999 |
| DPB1*04:02:01G | 4.55 | 1.61 | 0.526 | 2.905 | 0.350-24.144 | >0.999 |
| DPB1*05:01:01G | 50.65 | 45.16 | 0.465 | 1.246 | 0.690-2.252 | >0.999 |
| DPB1*09:01:01G | 1.30 | 1.61 | >0.999 | 0.803 | 0.071-9.015 | >0.999 |
| DPB1*10:01:01G | 0.00 | 1.61 | 0.287 | - | - | >0.999 |
| DPB1*13:01:01G | 5.84 | 0.00 | 0.117 | - | - | >0.999 |
| DPB1*135:01 | 1.30 | 1.61 | >0.999 | 0.803 | 0.071-9.015 | >0.999 |
| DPB1*14:01:01G | 2.60 | 4.84 | 0.412 | 0.524 | 0.114-2.415 | >0.999 |
| DPB1*17:01:01G | 2.60 | 3.23 | >0.999 | 0.800 | 0.143-4.484 | >0.999 |
| DPB1*19:01:01G | 1.30 | 1.61 | >0.999 | 0.803 | 0.071-9.015 | >0.999 |
| DPB1*21:01:01G | 1.30 | 0.00 | >0.999 | - | - | >0.999 |
| DPB1*26:01:02G | 0.65 | 0.00 | >0.999 | - | - | >0.999 |
| DPB1*28:01:01G | 0.65 | 0.00 | >0.999 | - | - | >0.999 |
| DPB1*47:01:01G | 0.65 | 0.00 | >0.999 | - | - | >0.999 |

Table S9 Comparison of select HLA alleles in HBV-infected ESLD patients, stratified by pre-operative HBV DNA level (positive vs. negative).

| Alleles | Frequency of patients with positive in pre-operative HBV DNA detection (%) (N=14) | Frequency of patients with negative in pre-operative HBV DNA detection (%) (N=63) | *P*-value | OR | 95% CI for OR | *Pc*-value |
| --- | --- | --- | --- | --- | --- | --- |
| DQA1*06:01 | 21.43 | 18.25 | 0.698 | 1.221 | 0.445-3.352 | >0.999 |
| DPB1*05:01:01G | 35.71 | 53.97 | 0.081 | 0.474 | 0.203-1.107 | >0.999 |
| DRB5 blank | 96.43 | 91.27 | 0.357 | 2.583 | 0.320-20.871 | >0.999 |

Table S10 Comparison of all HLA-DRB5 alleles between ESLD (n=108) and healthy controls (n=453) in the Zhejiang Han population when the blank was also counted.

| Alleles | Frequency of ESLD (%) | Frequency of healthy controls (%) | *P*-value | OR | 95% CI for OR | *Pc*-value |
| --- | --- | --- | --- | --- | --- | --- |
| DRB5*01:01 | 7.41 | 13.25 | 0.018 | 0.524 | 0.304-0.903 | 0.108 |
| DRB5*01:02 | 0.00 | 1.88 | 0.086 | - | - | 0.43 |
| DRB5*01:03 | 0.46 | 0.22 | 0.474 | 2.102 | 0.190-23.292 | >0.999 |
| DRB5*01:08N | 0.00 | 0.22 | >0.999 | - | - | >0.999 |
| DRB5*02:02 | 0.46 | 0.99 | 0.732 | 0.464 | 0.058-3.679 | >0.999 |
| DRB5*02:03 | 0.93 | 0.33 | 0.247 | 2.813 | 0.467-16.940 | 0.988 |
| DRB5 blank | 90.74 | 83.11 | 0.005 | 1.991 | 1.218-3.256 | 0.035 |

Table S11 Comparison of all HLA-DRB5 alleles between HBV-related ESLD (n=77) and healthy controls (n=453) in the Zhejiang Han population when the blank was also counted.

| Alleles | Frequency of HBV-related ESLD (%) | Frequency of healthy controls (%) | *P*-value | OR | 95% CI for OR | *Pc*-value |
| --- | --- | --- | --- | --- | --- | --- |
| DRB5*01:01 | 5.84 | 13.25 | 0.009 | 0.407 | 0.202-0.819 | 0.056 |
| DRB5*01:02 | 0.00 | 1.88 | 0.172 | - | - | 0.779 |
| DRB5*01:03 | 0.65 | 0.22 | 0.376 | 2.954 | 0.266-32.780 | >0.999 |
| DRB5*01:08N | 0.00 | 0.22 | >0.999 | - | - | >0.999 |
| DRB5*02:02 | 0.00 | 0.99 | 0.443 | - | - | >0.999 |
| DRB5*02:03 | 1.30 | 0.33 | 0.156 | 3.961 | 0.656-23.898 | 0.779 |
| DRB5 blank | 92.21 | 83.11 | 0.004 | 2.404 | 1.301-4.444 | 0.028 |

Table S12 Comparison of all HLA-DRB5 alleles between non-HBV-related ESLD (n=31) and healthy controls (n=453) in the Zhejiang Han population when the blank was also counted.

| Alleles | Frequency of non-HBV-related ESLD (%) | Frequency of healthy controls (%) | *P*-value | OR | 95% CI for OR | *Pc*-value |
| --- | --- | --- | --- | --- | --- | --- |
| DRB5*01:01 | 11.29 | 13.25 | 0.659 | 0.834 | 0.371-1.873 | >0.999 |
| DRB5*01:02 | 0.00 | 1.88 | 0.556 | - | - | >0.999 |
| DRB5*01:03 | 0.00 | 0.22 | >0.999 | - | - | >0.999 |
| DRB5*01:08N | 0.00 | 0.22 | >0.999 | - | - | >0.999 |
| DRB5*02:02 | 1.61 | 0.99 | 0.486 | 0.464 | 0.058-3.679 | >0.999 |
| DRB5*02:03 | 0.00 | 0.33 | >0.999 | - | - | >0.999 |
| DRB5 blank | 87.10 | 83.11 | 0.415 | 1.372 | 0.640-2.940 | >0.999 |

Table S13 Comparison of all HLA-DRB5 alleles between HBV-related ESLD (n=77) and non-HBV-related ESLD (n=31) in the Zhejiang Han population when the blank was also counted.

| Alleles | Frequency of HBV-related ESLD (%) | Frequency of non-HBV-related ESLD (%) | *P*-value | OR | 95% CI for OR | *Pc*-value |
| --- | --- | --- | --- | --- | --- | --- |
| DRB5*01:01 | 5.84 | 11.29 | 0.273 | 0.488 | 0.173-1.373 | >0.999 |
| DRB5*01:03 | 0.65 | 0.00 | >0.999 | - | - | >0.999 |
| DRB5*02:02 | 0.00 | 1.61 | 0.287 | - | - | >0.999 |
| DRB5*02:03 | 1.30 | 0.00 | >0.999 | - | - | >0.999 |
| DRB5 blank | 92.21 | 87.10 | 0.241 | 1.753 | 0.679-4.523 | >0.999 |

Table S14 Comparison of select HLA alleles in healthy controls by age group (>30 vs ≤30 years).

| Alleles | Frequency of healthy controls aged >30 years (%) (N=225) | Frequency of healthy controls aged ≤ 30 years (%) (N=228) | *P*-value | OR | 95% CI for OR | *Pc*-value |
| --- | --- | --- | --- | --- | --- | --- |
| DQA1*06:01 | 7.78 | 9.65 | 0.318 | 0.790 | 0.496-1.256 | >0.999 |
| DPB1*05:01:01G | 36.44 | 36.18 | 0.935 | 1.011 | 0.771-1.326 | >0.999 |
| DRB5 blank | 80.00 | 86.18 | 0.013 | 0.641 | 0.451-0.912 | 0.091 |
